# Facts from Correspondents

**Published:** 1846-03

**Authors:** 


### FACTS FROM CORRESPONDENTS.

---

### PECULIAR MORBID EFFECTS FROM QUININE.

We are indebted to Wm. H. Gardner, M.D., of Munfordsville, Ky., for the following communication. It may be a question whether it was the opium or the quinine that gave rise to the unpleasant symptoms in the cases related; but the cases are instructive, as showing that alarming effects sometimes attend the administration of these articles.

"I am in the habit of treating our simple autumnal intermittent and remittent fevers with an emetic, if called in the first instance, followed by a cathartic; and after evacuating the stomach and bowels fully, my practice is to arrest the paroxysm by quinine, opium, and ipecacuan combined. I give the quinine in doses varying from three to twelve grains, repeated every four or six hours, combined with from one to three grains of opium, and as much ipecac. This course I have found altogether satisfactory, pretty uniformly arresting the fever by the second or third day. I have never known any bad effects from the quinine when thus administered, except what seemed to be exhibited in the following cases:

Aug. 3d. Called to see Mrs. H., aged 60, of stout constitution; has had a regular paroxysm of fever every day during the last week; had a well marked chill this morning, followed by fever, which lasted nearly all day—she is now (evening) perspiring moderately. Has taken two doses of some purgative pills, which she says have acted well. Says she has enjoyed good health all her life, with the exception of an occasional attack of cramp colic, to which she has been subject many years. Says she cannot bear the least portion of opium or quinine; that she once took some powders which contained quinine and opium, and that they "were near killing her." To all this I was incredulous, and as the paroxysm was expected next morning, I

made the following prescription: ℞ Sulph. quinine, 16 grs., sulph. morphine, 2 grs., ipecac., 4 grs.—make into 8 pills with crumb of bread. I gave two of the pills at once, and sat down to wait their effects, intending to repeat every four hours. In twenty minutes after the pills were taken, she complained of slight nausea; in a few minutes more of cramp and pain at the stomach, which became so severe as to make her call for relief; and in less than 20 minutes from the time she first complained of the nausea, she was pulseless, speechless, and looked ghastly as death. She had a peculiar convulsive, gasping, retching, indicative of something very poisonous at the stomach. With much difficulty I poured down her a glass of warm water, and repeated it every few minutes with ipecac. dissolved till free vomiting was induced. The symptoms were now allayed to some extent for a time, but returned again with violence, and it was not till after vomiting a third or fourth time that she seemed to be permanently relieved, and went to sleep. Next morning she complained of severe soreness in the epigastrium, but missed her chill. A dose or two of calomel and rhubarb completed the cure.

I have seen a similar effect produced in only one other case—that of a young man to whom I gave 8 grs. of quinine and 10 grs. Dover's powder, to prevent a paroxysm which was expected in two hours. In a few minutes after the exhibition of the medicine most of the unpleasant symptoms enumerated in the above case came on, but not to so great an extent. Vomiting speedily induced by warm water and ipecac. relieved him.

It is proper, however, to add that I have since given this individual quinine and opium in large doses without any bad effect whatever.

Feb. 1846."

---

### THE AMERICAN JOURNAL OF SCIENCE AND ARTS.

We direct the attention of our readers to the prospectus of the *American Journal of Science and Arts,* which they will find on the cover. No periodical publication in America has done so much for the scientific reputation of our country as this Journal, and for this reason it has claims upon every man who loves his country. But it has other and peculiar claims upon all who love science. We trust it will find many subscribers among the readers of the Western Journal.

